# Risk and protective factors related to having a child with congenital heart diseases (CHD): a case-control study in Iran

**DOI:** 10.3389/fped.2023.1170743

**Published:** 2023-07-03

**Authors:** Mostafa Amini-Rarani, Salah Eddin Karimi, Neda SoleimanvandiAzar, Mehdi Nosratabadi

**Affiliations:** ^1^ Health Management and Economics Research Center, Isfahan University of Medical Sciences, Isfahan, Iran; ^2^ Social Determinants of Health Research Center, Health Management and Safety Promotion Research Institute, Tabriz University of Medical Sciences, Tabriz, Iran; ^3^ Preventive Medicine and Public Health Research Center, Psychosocial Health Research Institute, Iran University of Medical Sciences, Tehran, Iran; ^4^ Social Determinants of Health Research Center, Isfahan University of Medical Sciences, Isfahan, Iran

**Keywords:** congenital heart diseases (CHD), risk factors, protective factors, folic acid, tobacco, alcohol, Iran

## Abstract

**Background:**

The heart is the first fully developed organ in early pregnancy, especially in the first trimester of pregnancy, so any factor that contributes to heart failure is life-threatening. Thus, it is important to identify the risk and preventive factors related to this disease and to provide a scientific basis for the control, prevention, management and treatment of Child with Congenital Heart Diseases (CHD).

**Objectives:**

As the etiology of CHD is multifactorial, to identify the risk and preventive factors, this study aimed to investigate the factors related to CHD in Tehran, Iran.

**Methods:**

The present case-control study was performed on 600 people including 200 mothers of children with CHD. Simple random sampling was performed in 2020. The control group was matched with the case group, and the data were analyzed by SPSS software at a significance level of 0.5.

**Results:**

The results showed that low socioeconomic status, low education, history of abortion, smoking, alcohol consumption are risk factors, and consumption of folic acid, and prenatal care are the protective factors against CHD.

**Conclusion:**

According to the findings, our emphasis should be on preventive strategies, education of mothers and public health experts on the need for folic acid and pregnancy care, and cessation or reduction of alcohol and tobacco use, especially in families with low socioeconomic status and low level of education.

## Background

Congenital heart disease (CHD) is the most common congenital defect in the world, which refers to the abnormal growth of the heart and arteries. Although in the western world, progress, innovation and prenatal diagnosis play a major role and have significantly reduced the mortality figures and in southern Europe the incidence of CHD is lower than in northern Europe and Anglo-Saxon countries (1), but this disease accounts for 1% of all live births and one-third of all congenital abnormalities (2), 94% of which occur in developing countries (3). In some studies in developing countries, the prevalence of CHD has been reported at 4–12 cases per 1,000 live births (4–6). CHD is the cause of 20%–30% of infant mortality and 20% of stillbirths (3). It is also a major global economic health threat (7) so in 2012, hospital costs for children with CHD were estimated to be higher than $ 6 billion (8). In 2021 in China, the cost of pediatric CHD was estimated to be $12.6 billion (5). Despite the stillbirths and infant deaths, even with improved prognosis and quality of life in CHD children using innovative medical techniques, congenital abnormalities are still the main causes of lifelong mental and physical disability in children, which can have a significant impact on their families and societies (3, 9).

The heart is the first fully developed organ in early pregnancy, especially in the first trimester of pregnancy, so any factor that contributes to heart failure is life-threatening. Although the main causes of CHD abnormalities remain unclear, it is obvious that 80% of the CHD cases are due to multiple environmental and genetic factors or the interaction between the two (10). It seems that an embryo that is genetically exposed to environmental stimuli may develop morphogenetic abnormalities of the heart during intrauterine development. In fact, the interaction between genetic predisposition and exposure to environmental stimuli may lead to congenital heart disease (11).

Various studies have linked factors, such as alcohol use, sex hormones, occupational exposure to rubella virus, maternal diabetes and the use of certain drugs, including thalidomide, lithium and folic acid antagonists, to varying degrees of CHD (11–13). Strengthening protective strategies such as avoiding autogenous substances, infection screening and treatment of the mother and her proper diet, including vitamins, and folic acid (14), can control about 70% of cases of this disease (15). Therefore, we can say that the etiology of CHD is multifactorial and in this regard, identifying the risk and preventive factors of this disease is important and necessary in order to provide a scientific basis for control, prevention, management and treatment of CHD. Thus, the aim of this study was to identify the risk and preventive factors related to CHD in Tehran, Iran.

## Methods

### Study design

This case-control study was carried out in 2020 on the mothers of children with CHD who were admitted to the Shahid Rajaie Cardiovascular Medical and Research Center in Tehran, Iran. This hospital is one of the largest cardiac referral hospitals in Asia and admits patients with cardiovascular diseases from various parts of Iran. Pediatric heart care is one of the most important clinical services offered in this hospital. The pediatric cardiology department, as a well-known and best-equipped clinic, provides educational, treatment, and research services for children and adolescents with heart diseases.

Inclusion criteria were: confirmed diagnosis of the child with CHD, in terms of Diagnosis during pregnancy (fetal echocardiography) and congenital heart disease diagnosed shortly after birth, and children under 5 years of age were included in this study.

Exclusion criteria were: Children with non-congenital heart disease, Children with congenital heart disease whose mothers have died, and Children who had non-Iranian nationality.

### Data collection

The data were collected primarily from mothers who attended the pediatric cardiology department between March 2020 and May 2021. Two hundred mothers of children with CHD (Congenital Heart Disease) were selected through simple random sampling. At first, a list of children with congenital heart disease was prepared; a random number was assigned to each child using Excel software. Then, we selected the samples with random numbers from 1 to 200. Finally, the checklist was completed by the mother of the selected child and allocated to the case group. For selecting the controls, a list of children who did not have congenital heart disease was prepared. Then, similar to the case group selection method, random numbers from 1 to 400 were selected using the simple random sampling method by Excel software.

According to the matching criteria, such as mother's age, child's age, child's gender and city of residence, 400 mothers of children without CHD were recruited and allocated to the control group (the ratio of cases to control groups was set to be 1:2).

The CHD diagnosis was made by a cardiologist. Registering the child as a child with congenital heart disease in the medical record. Besides, for more confirmation and certainty, the mother was also asked whether her child had this disease at birth or not. It should be noted that fetal echocardiography records or the child's cardiologist's report are available as documentation of congenital heart disease in the medical report.

Mothers in case and control groups were asked to fill the study checklist. The explanatory variables were selected based on the WHO framework regarding social determinants of health presented by Solar and Irwin (16).

### Definition of variables

CHD was chosen as a dichotomous outcome variable (mothers with CHD children or not). The polychoric principal component analysis (PCA) was used to construct the households' socioeconomic status (17). One of the assumptions underlying the classic PCA is that, the input variables are normal. However, as our data regarding the households' socio-economic status were discrete (including binary and ordinal variables), this assumption was clearly violated. As a result, the polychoric PCA was used in this study. The following variables were used in the polychoric PCA model: The mother's education level, father's education level, father's occupation, house ownership, owning a personal computer, and having a kitchen, a bathroom, a vacuum cleaner, a washing machine, and a freezer. Accordingly, five socioeconomic quintiles including the lowest, low, moderate, high, and highest quintiles were identified and used in the subsequent analysis.

The social variables included; the socio-economic status of the household, level of education of mother and father, occupation of mother and father, nationality, place of residence, number of children and family marriage. The medical/biological variables included; the mother's age at delivery, father's age, the number of childbirth deliveries, receiving pregnancy health care, history of abortion, and chronic disease, and the use of folic acid. Also, the lifestyle variables included physical activity (Having or not having physical activity), alcohol consumption, and smoking (whether the mother used alcohol or smoking during pregnancy or not).

### Data analysis

Descriptive analysis was conducted in terms of frequencies and percentages. Given that CHD was a binary outcome variable, the Chi-square test and the logistic regression were used to identify the main explanatory variables. To understand the real factors associated with CHD, the significant variables detected through the Chi-square test were first identified and then entered into a multivariate logistic regression model using the forward method. The cutoff point for univariate binary logistic regression analysis was set at ≤0.2. Also, the Hosmer–Lemeshow test was used to examine the goodness of fit for the logistic model against actual outcomes. The results of the Hosmer–Lemeshow test did not show a significant difference/relationship between the variables (*p* > 0.05), suggesting an adequate overall fit for the model.

### Ethical considerations

The current study was approved by the Research Ethics Committee of Isfahan University of Medical Sciences, Isfahan, Iran (IR.MUI.RESEARCH.REC.1399.118). All participants were informed regarding the aim and objectives of the study, and written informed consent was obtained from all of the participants prior to participation.

## Results

We analyzed the data of 600 mothers in 2020–21, of whom 33.3% (mothers who had children with CHD) and 66.7% (mothers who had children without CHD) were investigated as the case and control groups, respectively. Most mothers in the control group had a moderate socioeconomic status (SES), whereas most mothers in the case group had the lowest SES. In the case group, most mothers were 18–28-year-old (44.5%), illiterate (32%), housewives (81.5%), and also had two children (43.5%) and had a history of abortion (57%). In the control group, most mothers were 29–39 years old (56.8%), and had a secondary school education (27%) and had no history of abortion (93.5%). Also, having a chronic disease, alcohol use and smoking was more prevalent among mothers with CHD children in comparison with the control group (Table 1).

**Table 1:** Descriptive characteristics of mothers with CHD children in the case and control groups referred to the heart hospital.

Variable		Frequency (%)			
		Case (*n* = 200)	Control (*n* = 400)	Total (*n* = 600)	*p*-Value^a^
Socio-economic status	Lowest	98 (49.0)	22 (5.5)	120 (20.0)	<0.001
	Low	68 (34.0)	52 (13.0)	120 (20.0)	
	Moderate	20 (10.0)	118 (29.5)	138 (23.0)	
	High	13 (6.5)	96 (24.0)	109 (18.2)	
	Highest	1 (0.5)	112 (28.0)	113 (18.8)	
Mother's education	Illiterate	64 (32.0)	18 (4.5)	82 (13.7)	<0.001
	Primary school	49 (24.5)	62 (15.5)	111 (18.5)	
	Secondary school	36 (18.0)	108 (27.0)	144 (24.0)	
	High school	38 (19.0)	105 (26.3)	143 (23.8)	
	University	13 (6.5)	107 (26.8)	120 (20.0)	
Father's education	Illiterate	20 (10.0)	13 (3.2)	33 (5.5)	<0.001
	Primary school	36 (18.0)	15 (3.8)	51 (8.5)	
	Secondary school	40 (20.0)	59 (14.7)	99 (16.5)	
	High school	55 (27.5)	118 (29.5)	173 (28.3)	
	University	49 (24.5)	195 (48.8)	244 (40.7)	
Mother's occupation	Housewife	163 (81.5)	205 (51.2)	368 (61.3)	<0.001
	Office worker	14 (7.0)	123 (30.8)	137 (22.8)	
	Non-office worker	23 (11.5)	72 (18)	95 (15.8)	
Father's occupation	Office worker	37 (18.5)	189 (47.2)	226 (37.7)	<0.001
	Non-office worker	152 (76.0)	181 (45.3)	333 (55.5)	
	Unemployed	11 (5.5)	30 (7.5)	41 (6.8)	
Nationality	Iranian	187 (93.5)	389 (97.3)	576 (96.0)	0.02
	Non-Iranian	13 (6.5)	11 (2.7)	24 (4.0)	
Place of residence	Urban	127 (63.5)	367 (91.8)	494 (82.3)	<0.001
	Rural	73 (36.5)	33 (8.2)	106 (17. 7)	
Number of children	One child	78 (39.0)	124 (31.0)	202 (33.7)	<0.001
	Two children	87 (43.5)	174 (43.5)	261 (43.5)	
	≥3 children	35 (17.5)	102 (25.5)	137 (22.8)	
History of having family marriage	Yes	83 (41.5)	61 (15.2)	144 (24.0)	<0.001
	No	117 (58.5)	339 (84.8)	456 (76.0)	
Mother's age at delivery time	15–17 (year)	10 (5.0)	0 (0.0)	10 (1.7)	<0.001
	18–28 (year)	89 (44.5)	113 (28.2)	202 (33.7)	
	29–39 (year)	82 (41.0)	227 (56.8)	309 (51.5)	
	≥40 (year)	19 (9.5)	60 (15.0)	79 (13.1)	
Father's age	17–25 (year)	33 (16.5)	13 (3.2)	46 (7.6)	<0.001
	26–30 (year)	37 (18.5)	68 (17.5)	105 (17.5)	
	31–35 (year)	59 (29.5)	76 (19.0)	135 (22.5)	
	36–40 (year)	33 (16.5)	138 (34.5)	171 (28.5)	
	≥41 (year)	38 (19.0)	105 (26.3)	143 (23.8)	
Number of childbirth deliveries	1	55 (27.5)	116 (29.0)	171 (28.5)	0.43
	2	83 (41.5)	180 (45.0)	263 (43.83)	
	>=3	62 (31.0)	104 (26.0)	166 (27.7)	
Receiving pregnancy health care	Received	101 (50.5)	377 (94.3)	478 (79.7)	<0.001
	Not received	99 (49.5)	23 (5.7)	122 (20.3)	
History of abortion	Yes	114 (57.0)	26 (6.5)	140 (23.3)	<0.001
	No	86 (43.0)	374 (93.5)	460 (76.7)	
History of Chronic disease	Yes	119 (32.5)	76 (19.0)	195 (32.5)	<0.001
	No	81 (40.5)	324 (81.0)	405 (67.5)	
Using folic acid	Yes	101 (50.5)	381 (95.2)	482 (80.3)	<0.001
	No	99 (49.5)	19 (4.8)	118 (19.7)	
Using alcohol	Yes	97 (48.5)	25 (6.2)	122 (20.3)	<0.001
	No	103 (51.5)	375 (93.8)	478 (79.7)	
Smoking	Yes	92 (46.0)	19 (4.7)	111 (18.5)	<0.001
	No	108 (54.0)	382 (95.3)	489 (81.5)	

^a^ Chi-square test.

To understand the real factors associated with CHD in children, all the significant variables in the chi-square test (except physical activity) were entered into a multivariate logistic regression model using the forward method. As shown In Table 2, the results of logistic regression revealed that among the variables with a statistically significant OR, the leading cause of CHD in children was related to the lowest SES quintile. The prevalence of CHD in the lowest and low SES group was 39.5 and 25.5 times higher than the highest SES group. Moreover, illiterate mothers and mothers with primary school education had 17.7 and 6.0 times higher odds of having children with CHD than mothers with university degrees, respectively. Having children with CHD was 13.9, 8.6 and 4.1 times higher in mothers with a history of smoking, alcohol use and abortion, respectively. In contrast, using pregnancy health care and folic acid had a protective effect on having children with CHD by up to 0.2 and 0.10 times, respectively.

**Table 2:** Factors related to CHD in children based on adjusted multivariate logistic regression.

Independent variables		OR^a^ (95% CI)	*p*-Value
Socio-economic status	Lowest	25. 5 (2.1–30.3)	0.01
	Low	39. 5 (3.1–49.4)	0.04
	Moderate	2. 3 (0.2–26.40)	0.51
	High	3. 9 (0.3–49.6)	0.28
	Highest	Reference	NA
Mother's education	Illiterate	17. 7 (1.4–22.4)	0.02
	Primary school	6.0 (1.9–39.0)	0.05
	Secondary school	1. 2 (0.2–6.2)	0.83
	High school	2. 7 (0.4–15.7)	0.27
	University	Reference	NA
Receiving pregnancy health care	Received	0. 2 (0.1–0.52)	0.004
	Not received	Reference	NA
History of abortion	Yes	4. 1 (1.3–13.3)	0.01
	No	Reference	NA
Using folic acid	Yes	0. 1 (0.1–0.6)	0.01
	No	Reference	NA
Using alcohol	Yes	8. 6 (2.0–37.0)	0.004
	No	Reference	NA
Smoking	Yes	13. 9 (2.7–42.8)	0.002
	No	Reference	NA
	Nagelkerke *R* square = 0.74		
	Hosmer–Lemeshow test: Chi-square = 5.46 L; *p*-value: 0. 70		

CI, confidence interval; NA, not applicable; OR, odds ratio.

^a^ Adjusted for mother's age, child's age, child's gender and city of residence.

According to the Nagelkerke *R* square, the study model explained 74% of the variance of having children with CHD. Moreover, the Hosmer–Lemeshow test was used to examine the goodness of fit for the logistic model against actual outcomes. The Hosmer–Lemeshow test suggested an adequate overall fit for the model (Table 2).

## Discussion

The aim of this study was to investigate the factors associated with having a child with CHD. The findings of this study statistically showed that low socioeconomic status, low education, history of abortion, smoking, and alcohol consumption, were the risk factors, and folic acid use and utilizing pregnancy health care were the protective factors against having a child with CHD.

The findings of the present study showed that low socioeconomic status is related to the odds of having a child with CHD. This finding is consistent with the study of Morales-Suárez-Varela et al. and Qu et al. (18, 19). The low socioeconomic status is not the risk factor for having a child with CHD in itself, but the circumstances in which the person lives, including the unfavorable living environment and exposure to various infectious and chemical substances, both at home and at work, increase the chances of having a child with CHD. Although several studies have concluded that having a job is associated with a better pregnancy (20, 21), other studies suggest that low socioeconomic status and work conflicts are associated with a risk of having a child with CHD (18, 22). Contrary to the results of the present study, the findings of a study by Egbe and colleagues showed that the incidence of CHD is lower in the lower socioeconomic classes (23). This discrepancy can be attributed to the unique features of the mentioned study, because they had only entered the diagnoses of CHD made during the hospital stay, into the study. Also, the mean age of patients at the time of diagnosis was 2 days. They had also conducted their study on specific ethnic groups such as Caucasians, and their study was a retrospective study that only had analyzed the available data. As a result, these findings may not have a high sensitivity. Finally, it seems that low socioeconomic status can be associated with fewer visits to health centers and facilities to receive health care services, resulting in less use of supplements and eventually the occurrence of diseases such as CHD (24, 25). It can be said that poor nutrition and low education are the results of living in the poverty among low socio-economic classes.

The present study showed that illiteracy and primary education (low education) increase the chances of having a child with CHD compared to higher education. This finding is consistent with the study of Kafian Atary et al., and Qu et al. (19, 26). Qu and colleagues in a study demonstrated that education for less than 12 years increases the odds of having a child with CHD. It can be said that people with low education are usually from the lower socio-economic classes of society, live in high-risk environments, and have less knowledge and awareness about using folic acid and utilizing prenatal health care, therefore they are more likely to have children with CHD than people with higher education.

The present study, in line with other studies, showed a significant relationship between the history of abortion and the increased risk of CHD (11, 27). The previous meta-analysis study showed that a history of abortion increased the risk of congenital miscarriage by 24% (27).

Our study found that mothers who consumed alcohol were 8.6 times more likely to give birth to children with CHD. In 1973, researchers found that alcohol consumption in mothers is a risk factor for heart diseases (28). Every year, more than 10,000 cases of CHD occur in the United States due to alcohol consumption (29). Consistent with the results of the present study, Fung et al. (30), Zhiyan Chen et al. (7), and Sudi Jemal et al. (3) found that alcohol consumption is associated with congenital heart disease so that consuming any amount of alcohol increases the risk of CHD (31). Although a study in Ethiopia (32) has shown that alcohol consumption is rarely associated with congenital anomalies, this finding should be treated with caution, as alcohol is able to be transmitted to the fetus through the placental membrane and therefore can have direct effects on the organogenesis of developing fetus (33, 34).In the present study, we found that smoking increases the chances of having a child with CHD. Studies have shown that smoking or exposure to chemicals substances makes mothers more susceptible to having a child with CHD (22). Other studies have shown that smoking in any way increases the chances of having a child with CHD (3, 26, 31, 32, 35, 36). The results of Nicole's study also indicated that in addition to the mother's smoking during pregnancy, her exposure to secondhand smoke and teratogens such as pesticides, metals and detergents, has a significant relationship with CHD (37). Another study by Deng and colleagues showed that fathers' smoking from the three months before pregnancy to the end of pregnancy increases the risk of having a child with CHD (38). Lijuan Zhao and colleagues also showed that active and passive smoking increases the risk of having a child with CHD (39).

In the present study, folic acid consumption was a protective factor against the development of congenital abnormalities, so it can prevent 90% of CHD cases. This finding is consistent with other studies (19, 37, 40, 41). Other studies have shown that the non-consumption of folic acid increases the chances of having a child with CHD (3). Iran's Ministry of Health has adopted policies through which, pregnant women would have free access to the folic acid supplement, but the lack of folic acid consumption in Iran may be due to low education, awareness, and ignorance of pregnant women and frontline healthcare professionals in regard to the importance of CHD and consumption of folic acid.

The findings of the present study indicate that prenatal health care is a protective factor against having a child with CHD so that it can prevent 80% of CHD cases. To the best of our knowledge, although there was no evidence to compare this finding with previous studies, it can be said that people who receive prenatal care, use folic acid, and are aware of the possibility of their children suffering from various diseases, keep themselves away from the risk factors. It is also possible that during pregnancy examinations, the child is diagnosed with CHD and her mother undergoes abortion, which reduces the chances of having a child with CHD in these women. Ultimately, it is possible that people who do not receive prenatal care live in financial and nutritional poverty, and this increases the risk of having a child with CHD in them.

It seems that the risk factors examined in the present study, such as receiving pregnancy health care, using folic acid, History of abortion, using alcohol, smoking, in interaction with each other and under the influence of important confounding variables of socioeconomic status and education level, this interaction are increase the risk of CHD, and would be need to be more investigated in future cohort and longitudinal studies.

## Strengths and limitations

Case-control studies are among the most reliable studies to analyze the relationship between the risk factors of a disease, but they are prone to recall bias, as mothers may be biased or erroneous in answering questions. Also, since in this study only mothers of live births infants in the hospital were included in the study, it is possible that selected bias has occurred in the study. As the evidence shows, these biases are inherent part of case-control studies, although it can be reduced to some extent during the interview by further explaining the variables and helping to remember them, but they cannot be completely eliminated. other possibility and bias is that non-CHD mothers are more prone to recall as well as choose to present a healthier lifestyle. In the present study, we are not investigated the relationship of gene pool in family marriages with CHD, and it is necessary to consider this variable in future studies. There are wide confidence intervals in several findings is another limitation of this study and a risk of low power. Therefore, care should be taken in generalizing the findings of the present study.

## Conclusion

Identifying CHD-related risk factors can help policymakers and stakeholders to reduce the prevalence of CHD by taking and strengthening preventive actions. Based on the findings, our emphasis is on preventive strategies, educating mothers and public health professionals about the need for folic acid and pregnancy health care, and stopping or reducing alcohol and tobacco use, especially in families with low socioeconomic status and low education levels.

## Data Availability

The raw data supporting the conclusions of this article will be made available by the authors, without undue reservation.
